# Effects of varying exercise intensities on muscle strength and depressive symptoms in Chinese adolescents: A 12-week randomized controlled trial

**DOI:** 10.1371/journal.pone.0336894

**Published:** 2025-11-21

**Authors:** Qing Chang, Xingrui Han, Wenlong Shen, Baoyi Yang, Zongrui Lin

**Affiliations:** 1 College of Physical Education and Health, East China Normal University, Shanghai, China; 2 Key Laboratory of Adolescent Health Assessment and Exercise Intervention, Ministry of Education, Shanghai, China; 3 Sports Department, Nanchang Haitang Middle School, Nanchang, Jiangxi, China; Japanese Academy of Health and Practice, JAPAN

## Abstract

The present study investigates the effects of physical education programs with varying exercise intensities on muscle strength and depressive symptoms in adolescents, while also evaluates the role of muscle strength improvement in alleviating depressive symptoms. A total of 266 eighth-grade students were divided into three groups based on exercise intensity (low, moderate, and vigorous). Over 12 weeks, students participated in physical education sessions three times per week. Muscle strength was assessed using the standing long jump and handgrip strength tests, while depressive symptoms were measured using the Center for Epidemiologic Studies Depression Scale (CES-D). Paired t-tests and one-way ANOVA were used to evaluate intra-group and inter-group differences, respectively. Multiple linear regression analysis was conducted to examine the predictive effect of muscle strength changes on depressive symptoms. Results indicated Significant improvements in standing long jump and grip strength were observed across all groups, with the vigorous-intensity group achieving the greatest gains. Depressive symptoms improved significantly in the moderate- and vigorous-intensity groups, with the latter showing the most pronounced reductions. Notably, improvements in standing long jump significantly predicted reductions in depressive symptoms, particularly in the vigorous-intensity group, whereas handgrip strength did not. These results suggest that integrating higher-intensity physical activities into school programs may have beneficial effects on both physical and mental health. Lower-limb muscle strength improvements predict reduced depressive symptoms, underscoring the importance of targeted strength training in physical education. This clinical trial has been registered with the Chinese Clinical Trial Registry under the registration number ChiCTR2500103874.

## Introduction

Adolescence is a pivotal period for both physical and psychological development. Insufficient physical activity during this stage has been linked to decreased muscle strength and an increased risk of mental health problems. Muscle strength is foundational for physical qualities such as endurance and coordination, while also contributing to overall skeletal and cardiovascular health [1]. Concurrent with these developments, mental health issues, such as depression, are prevalent among adolescents due to academic pressures and social challenges. Recent studies have highlighted a depression prevalence rate of approximately 24.3% among Chinese middle school students [2], with severe cases reaching 8.9% [3]. Depression undermines learning efficiency and quality of life, and may escalate into severe psychological issues, including self-harm or suicidal behavior [4]. Therefore, exploring scientifically effective physical education models to improve the physical and mental health of middle school students has become an urgent research priority.

While the benefits of physical exercise for mental health are well-documented [5], the specific effects of varying exercise intensities on depressive symptoms and muscle strength development remain under-explored. The question of optimal exercise intensity continues to be debated among researchers, with studies producing inconsistent findings regarding which levels best promote physical and psychological health [6,7]. Exercise intensity is a critical factor influencing the effectiveness of physical activity. Low-intensity exercises, while less demanding and more accessible, may fail to stimulate sufficient physiological changes to have a meaningful impact on mental health [8]. Conversely, while vigorous-intensity exercise may be more effective in enhancing muscle strength, it may not be sustainable for all individuals, particularly those with lower baseline fitness levels or pre-existing psychological conditions [9]. To address this gap, the present study uniquely investigates the differential effects of varying exercise intensities (low, moderate, and vigorous) on adolescents’muscle strength and depressive symptoms. Unlike previous research, which often generalizes the benefits of exercise, this study aims to identify the optimal intensity required for significant improvements in both muscle strength and depressive symptom outcomes. By focusing on exercise intensity as a variable, this study aims to provide nuanced insights into designing tailored physical education programs.

Research indicates that reduced muscle strength is associated with an increase in depressive symptoms. Sun’s studies have demonstrated a negative correlation between grip strength and depressive symptoms, with lower levels of grip strength being associated with a higher incidence of depressive symptoms. Conversely, higher grip strength has been identified as a protective factor against depression [10]. The study conducted by Van Milligen BA et al. in adults demonstrated that individuals with depressive symptoms exhibit significantly lower muscle strength compared to healthy controls [11]. However, the extent to which changes in muscle strength can predict variations in depressive symptoms remains unknown. Therefore, the potential of muscle strength as a predictor for mental health outcomes, particularly depressive symptoms, warrants deeper investigation. Understanding the relationship between these variables can inform interventions designed to enhance muscle strength as a potential protective factor against the development of depression.

To fill this gap, we conducted a 12-week randomized controlled trial (RCT) to examine the effects of different exercise intensities on both muscle strength and depressive symptoms among adolescents, as well as to investigate the predictive role of muscle strength improvements in reducing depressive symptoms. By providing insights into the efficacy of varying exercise intensities and the potential of muscle strength as a predictive factor for depressive symptoms, this research seeks to guide the development of targeted physical education interventions that promote comprehensive adolescent health.

## Materials and methods

### Participants

The sample size was determined using G*Power version 3.1 to ensure sufficient power to detect medium-sized effects in the statistical analyses [12]. Based on prior literature examining exercise interventions on depressive symptoms and muscle strength, a medium effect size (f² = 0.25) was anticipated [13–15]. This corresponds to an expected group difference equivalent to approximately 0.5 standard deviations in primary outcomes such as depressive symptom scores and standing long jump performance. With an alpha level of 0.05 and a desired power of 0.80, the required sample size was calculated to be 158 participants. To account for potential attrition, we recruited a larger sample.

Participant recruitment occurred from September 6, 2024, to September 13, 2024. Follow-up assessments took place after the 12-week intervention, with the final post-intervention evaluations completed in January 2025. All participants were informed about the study and provided consent to participate. The study involved 303 eighth-grade students from a school located in Jiangxi Province, China. To actively recruit participants, invitation letters and consent forms were sent to school principals. After they reviewed the research protocol and approved the study, we invited physical education teachers to participate in the recruitment process and attend training sessions on study-related assessments and the physical education teaching program.

Students were eligible participants if they met all inclusion criteria, including: (a) enrollment in Grade 8; (b) typical physical, cognitive, and mental development without impairments; and (c) provision of consent/assent to complete all study-related questionnaires and tests. Students were excluded if they (a) declined to participate; (b) failed to complete either the tests or the questionnaire; or (c) had severe physical, cognitive, or mental impairments, disabilities, accidents, injuries, or illnesses.

Based on the inclusion and exclusion criteria, a final sample of 266 participants was included in the study. Students and their parents provided written informed consent, which detailed the purpose, procedures, and precautions of the study, on the assessment day. After signing the consent form, participants submitted basic demographic and background information. Participants were assured that their personal data would be kept confidential, and that any information collected would be used solely for the purpose of this research. All data were anonymized and stored securely, in compliance with data protection regulations. The study was approved by the Research Ethics Commission of East China Normal University, under approval letter number (2024) No.17. This clinical trial has been registered with the Chinese Clinical Trial Registry under the registration number ChiCTR2500103874. All ongoing and related trials for this intervention are also registered. The initial study protocol was classified as a routine school-based physical education program evaluation. Upon completion of the study, the research team recognised that the study design met the World Health Organization’s definition of a clinical trial. Registration with the Chinese Clinical Trial Registry was therefore completed retrospectively, before any data analysis or unmasking of group allocation. The authors regret this procedural delay and confirm that no protocol deviations occurred between enrolment and registration.

### Experimental design

Participants were randomly assigned to one of three groups (low-, moderate-, or vigorous-intensity exercise) using a computer-generated block randomization sequence (block size = 6). Group allocation was performed by a researcher who was not involved in the intervention delivery or outcome assessment. Given the nature of the intervention, blinding of participants and physical education teachers was not feasible; however, outcome assessors and data analysts were blinded to group assignments to minimize bias.

This study employed a two-factor experimental design, with exercise intensity levels (low, moderate, and vigorous) as the independent variable, and muscle strength (assessed through standing long jump and grip strength) as well as depressive symptoms as the dependent variables. In the second phase of analysis, multiple linear regression was conducted to examine the predictive relationships between muscle strength changes and the alleviation depressive symptoms, with separate regression models developed for lower-limb strength (standing long jump) and upper-limb strength (grip strength). This approach allowed for a more detailed examination of how improvements in specific muscle groups influenced mental health outcomes across the three intensity conditions. Participants in all groups engaged in volleyball-based training sessions, each lasting 40 minutes, as part of a 12-week physical education program conducted three times per week.

### Intervention

Physical education teaching was conducted using the Health and Physical Education Curriculum Model of China [16]. The time allocation for the Chinese Health and Physical Education Curriculum Model was as follows: the warm-up phase was 7 minutes (1 minute for classroom routine and 6 minutes for preparatory activities), the main phase was 30 minutes (20 minutes for motor skills training and 10 minutes for physical fitness exercises), and the cool-down phase was 3 minutes (2 minutes for relaxation activities and 1 minute for a class summary). The specific teaching requirements of the Chinese Health and Physical Education Curriculum Model are presented in Table 1.

**Table 1:** The physical education teaching content of Health and Physical Education Curriculum Model of China.

Intervention content		
General requirements	Teaching objective	Improve the students’ level of physical and mental health.
	Teaching method	Advocates the change from teaching-oriented to learning-oriented. The key to realizing this change is to advocate for diversified teaching methods.
	Teaching contents	What educators advocate is that students are willing to learn, which helps to promote the students’ physical and mental health. No matter if it is formal competitive sports or folk sports events, as long as the students like to learn in class and put it into practice, which should become the content of teaching.
	Teaching atmosphere	It highlights the pleasant interactions between teachers and students in the classroom with their full and high emotions, lively and warm scenes, and positive atmosphere.
	Learning evaluation	Process evaluation and result evaluation together for students.
Three key points	Exercise load	The exercise intensity is measured by the average heart rate of all students, categorized into three groups: The average heart rates per physical education class were 100–120 bpm for the low-intensity exercise group, 120–140 bpm for the moderate-intensity exercise group, and 140–160 bpm for the vigorous-intensity exercise group.
	Diverse physical fitness exercises	Diversified physical fitness training means that each physical education class should have 10 minutes of physical fitness training, which should focus on diversification, fun, and compensation.
	Structural motor skills	Structural motor skills refers to each physical education class’s motor skills learning time with a 20-minute guarantee. Both a single skill and combination skills of learning and practice, more to the single skill and combination skills of learning and practice and complete activities or competition organic connection, paying more attention to take good advantage of and improve the skill when you use it.

Table 1 The Health and Physical Education Curriculum Model of China is structured around three key points and five general requirements. The three key points focus on exercise load, diverse physical fitness exercises, and structural motor skills development. Exercise intensity is adjusted according to heart rate, with the groups categorized as low, moderate, or vigorous intensity, corresponding to heart rate ranges of 100-120 bpm, 120-140 bpm, and 140-160 bpm, respectively. The five general requirements include clear teaching objectives aimed at improving students’ physical and mental health, diversified teaching methods, an engaging and positive classroom atmosphere, an emphasis on student-driven learning, and a dual approach to learning evaluation that includes both process and outcome assessments. The intervention for each group involves identical physical activities, with the primary difference being the intensity of exercise, which is adjusted by modifying the duration of rest intervals and the complexity of tasks performed during the training sessions.

The main exercise segment involved volleyball-based skill training (e.g., forearm passes, overhand serves, agility-based movement drills) and diverse physical fitness exercises (e.g., squats, lunges, planks). These were standardized across all groups, with exercise intensity as the only manipulated variable.

Exercise intensity was assessed using heart rate as the primary parameter [17]. In this study, exercise intensity refers to the average heart rate of students recorded throughout a single physical education class. During the instructional period, students wore Polar heart rate monitors (Polar Team OH1, 6248A-2L) to record their heart rates and monitor exercise intensity throughout the class [18]. Prior to testing, the participants’ basic information (full name, sex, date of birth, resting heart rate, and age-predicted maximal heart rate) was entered into the Polar system, and the device was positioned on the left upper arm. Heart rate data were continuously recorded throughout each session and synchronized with the Polar Team application.

The intensity classifications were as follows: Low intensity: 100–120 bpm (~40% VO_2_max), Moderate intensity: 120–140 bpm (~56% VO_2_max), Vigorous intensity: 140–160 bpm (~71% VO_2_max). The low-intensity group performed activities with longer and more frequent rest periods (e.g., 30–60 seconds between drills) and simplified task execution, while the moderate-intensity group employed moderate rest intervals (e.g., 15–30 seconds) and standard task complexity; in contrast, the vigorous-intensity group engaged in sessions characterized by minimal rest intervals (e.g., 0–15 seconds), increased repetitions, and enhanced task complexity—such as faster-paced drills and combined skill sequences—to achieve and sustain elevated heart rates.

Participants’ compliance was verified by calculating the percentage of sessions in which average heart rate fell within the target zone. Participants missing more than 15% of sessions or failing to meet intensity targets were excluded from final analysis.

To promote fidelity, all instructors received standardized training and followed a fixed lesson plan with embedded intensity adjustments (e.g., rest intervals, task complexity) appropriate to the assigned group. All sessions were conducted during the same time of day (either all in the morning or all in the afternoon within a given day) to minimize circadian influences on performance and mood. The data collection team, which was blinded to group assignments, ensured that participants’ physical and mental health metrics were consistently measured across all time points.

### Design rationale for exercise intensities

According to the study by Ekelund et al., in adolescents aged 14–15 years, heart rates of 120, 140, and 160 correspond to 40.1%, 55.6% and 71.1% of peak oxygen uptake (VO_2_max), respectively. The exercise intensities selected in this study were HR_100–120_, HR_120–140_, and HR_140–160_, representing low-intensity, moderate-intensity, and moderately vigorous-intensity exercise, respectively [19].

Low-intensity exercise: This range was chosen to represent minimal cardiovascular and muscular load [20]. This intensity is accessible to participants with lower baseline fitness levels, serving as an entry point for physical activity. While less demanding, it ensures participation without imposing excessive strain, particularly for adolescents new to structured exercise programs. However, its limited physiological stimulus is hypothesized to result in comparatively fewer benefits in muscle strength and mental health outcomes [21].

Moderate Intensity: This range supports cardiovascular and muscular adaptations while maintaining sustainability for most participants [17]. Moderate-intensity exercise has been extensively linked to improvements in mental health, including reductions in depressive symptoms, due to its capacity to elicit beneficial neurochemical changes such as increased levels of brain-derived neurotrophic factor (BDNF) and decreased cortisol secretion [22].

Vigorous-intensity: Vigorous-intensity exercise has been shown to induce significant neuromuscular adaptations, increasing muscle hypertrophy and strength [23]. It also promotes pronounced neurochemical changes associated with improved mood and mental health outcomes, including elevated BDNF levels and reduced systemic inflammation [24]. Despite its potential benefits, this intensity may not be suitable for all individuals due to its higher physical demands, necessitating careful participant monitoring.

These intensity ranges collectively provide a comprehensive framework to evaluate the differential impacts of physical education programs on adolescents’ muscle strength and mental health. By incorporating these benchmarks, the study aims to elucidate the optimal intensity range for achieving significant improvements in physical and psychological outcomes.

### Outcome measures

#### Assessment of muscle strength.

The evaluation of muscle strength includes assessments of both upper and lower limb strength. Grip strength is measured to evaluate upper limb strength, while the standing long jump test assesses lower limb strength [25].

Grip strength is measured using a handgrip dynamometer (EH101, Camry, China) [26]. The participant stands upright, holding the dynamometer in their dominant hand with the arm extended downward and slightly away from the body. They are instructed to exert maximum force by squeezing the device as firmly as possible for a duration of 3–5 seconds. This measurement is repeated three times, with brief rest intervals between trials, and the highest value is recorded as the final result.

The standing long jump test is conducted using a jump mat with a marked scale. Participants stand behind a designated line and jump as far forward as possible from a stationary position. The distance is measured from the take-off line to the nearest point of contact (usually the heels). Each participant is allowed three attempts, with the longest distance recorded as the final result. Greater distances indicate better lower limb power.

#### Assessment of depression.

Depression was assessed using the 20-item Chinese version of the Center for Epidemiologic Studies Depression Scale (CES-D) [27]. Participants rated the occurrence of depressive symptoms experienced over the preceding week using a 4-point Likert scale, where 0 represented “not at all” and 3 indicated “a lot”. Depression scores were calculated by summing the responses to the 20 items, with potential scores ranging from 0 to 60. Higher scores indicated more severe depressive symptoms. The internal consistency of this scale was acceptable in this study [28]. This confirms that the CES-D is a reliable and valid tool for capturing depressive symptoms in this population.

Students completed the questionnaires under the supervision of the researcher and teachers to ensure uniformity in administration. The assessments were conducted during school hours, with clear instructions provided. No time limit was imposed, and students could ask for clarification if needed. Teachers received prior training to ensure consistent administration across the three participating groups. To minimize external influences, the questionnaires were distributed and collected on the same day.

#### Potential cofounders.

Several confounding factors, including age, sex, body mass index (BMI), extracurricular physical activity, registered residence, only child, parental education level, and family life pattern, were considered in this study. Participants’ body weight and height were measured using a height-weight meter (HGM-300, Henan, China). We ensured that all participants wore light clothing and were without shoes during measurements. Each measurement was taken twice and recorded to the nearest 0.1 kg/cm. BMI was calculated as Weight(kg) / Height^2 (m^2).

Extracurricular physical activity was monitored using accelerometer (ActiGraph, GT3X+) [29]. Twenty students from each class were selected as participants and instructed to wear the device for one week to assess their physical activity during this period. Prior to monitoring, participants’ basic information (including name, gender, date of birth, height, and weight) was entered into the accelerometer. Participants were provided with guidance on properly wearing the device, including instructions on duration (24 hours per day, except during bathing) and placement (right hip). The accelerometer was set to a sampling frequency of 30 Hz with a 5-second epoch interval.

The registered residence was categorized as either urban or rural. Parental education levels were classified as low, medium, or high. Family life patterns were divided into four types: 1) living only with parents, 2) living with parents and grandparents, 3) single-parent families, and 4) remarried families.

### Data analysis

Data analyses were performed using IBM SPSS Statistics Version 26.0 (IBM SPSS Statistics for Windows, Version 26.0, Armonk, NY, USA). Normality of distribution for each continuous variable was assessed using both statistical tests (Kolmogorov-Smirnov test) and graphical methods (normal probability plots). Multicollinearity was examined using the Variance Inflation Factor (VIF). The descriptive statistics of continuous variables are presented as means and standard deviations (mean ± SD), while categorical variables are reported as absolute numbers and proportions. Probability distributions of categorical variables across the three groups were analyzed using the Chi-square test. Statistical comparisons of clinical outcomes between groups at baseline were performed using analysis of variance or the Kruskal–Wallis test as appropriate. Paired samples t-tests were used to assess within-group changes in standing long jump, handgrip strength, and depressive symptoms. For between-group comparisons, one-way analysis of variance (ANOVA) was utilized to determine significant differences among the three groups regarding changes in these variables.The differential impact of teaching interventions on outcomes was assessed by analyzing the pre-post difference scores for each variable using one-way ANOVA, followed by post hoc comparisons with Bonferroni adjustments to pinpoint group-specific effects. To evaluate whether improvements in muscle strength predict reductions in depressive symptoms, multiple linear regression analyses were conducted. Independent variables included changes in standing long jump and handgrip strength, while the dependent variable was the reduction in depressive symptoms. Covariates including gender, age, BMI, and extracurricular physical activity levels were adjusted for in the regression models. Statistical significance was set at p < 0.05.

## Results

### Flow of participants through the study

**Fig 1:**
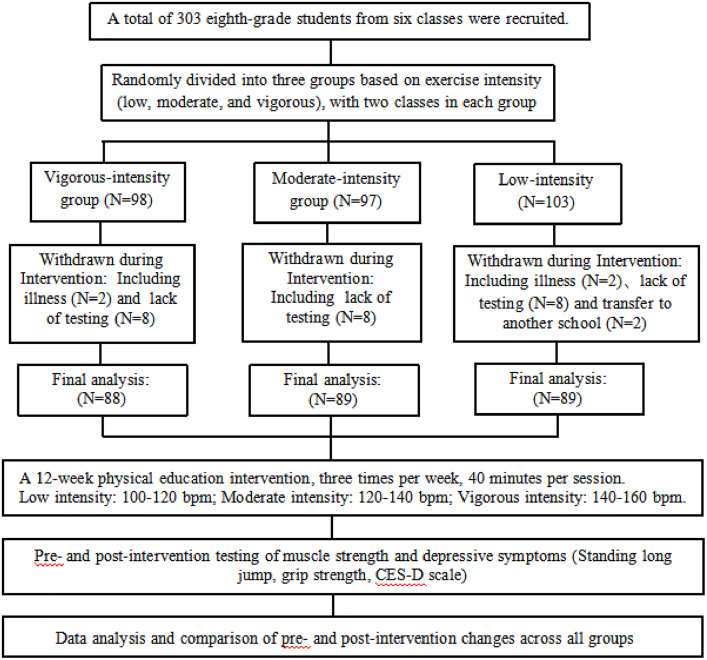
CONSORT flowchart.

The study involved 303 junior school students from six classes located in Jiangxi province, China. A total of 266 students (142 boys and 124 girls) were eligible for this study (S1 Dataset). All the students from the six classes were informed about the project. They received written informed consent forms to be completed by their parents, and they were informed that their participation in the study was part of their normal classes. Participants were enrolled in the study and randomized into the low-intensity, moderate-intensity, and vigorous-intensity groups. Fig 1 shows the CONSORT flowchart.

### Participant characteristics

Participant characteristics are presented in Table 2. The distributions of age, BMI, registered residence, and family life pattern were similar among the groups (p > 0.05). However, significant differences were observed in gender distribution (p = 0.019), father education level (p = 0.024), maternal education level (p = 0.019), and the proportion of only children (p = 0.004). These results indicate overall group comparability, with notable differences in certain socioeconomic and demographic factors.

**Table 2:** Demographic characteristics of participants.

Variables	Vigorous-intensity group (N = 88)	Moderate-intensity group(N = 89)	Low-intensity group (N = 89)	P value
Age (mean, SD)	14.09 (0.47)	14.06 (0.32)	14.08 (0.35)	0.829
BMI (mean, SD)	20.16 (3.57)	20.19 (3.69)	20.20 (3.11)	0.996
Gender, n (%)				
Boys	44 (50.0)	58 (65.1)	40 (45.0)	0.019
Girls	44(50.0)	31(34.9)	49 (55.0)	
Registered residence, n (%)				
Urban	19 (21.5)	27 (30.3)	30 (33.7)	0.162
Rural	69 (78.4)	62 (69.6)	59 (66.3)	
Only child, n (%)				
Yes	1 (1.1)	10 (11.2)	2 (2.2)	0.004
No	87 (98.9)	79 (88.8)	87 (97.8)	
Father education level, n (%)				
Low Education Level	45 (51.1)	57 (64.0)	38 (42.7)	0.024
Middle Education Level	25 (28.4)	16 (18.0)	21 (23.6)	
High Education Level	18 (20.5)	16 (18.0)	30 (33.7)	
Maternal education level, n (%)				
Low Education Level	49 (55.7)	56 (62.9)	39 (43.8)	0.019
Middle Education Level	26 (29.5)	19 (21.3)	22 (24.7)	
High Education Level	13 (14.8)	14 (15.7)	28 (31.5)	
Family life pattern, n (%)				
Living only with parents	49 (55.7)	56 (62.9)	54 (60.7)	0.756
Living with parents and grandparents	35 (39.8)	27 (30.3)	31 (34.8)	
Single-parent families	3 (3.4)	5 (5.6)	2 (2.2)	
Remarried families	1 (1.1)	1 (1.1)	2 (2.2)	
Depressive symptoms	16.39 (9.24)	13.75 (7.96)	14.00 (12.20)	0.154
Standing long jump/(cm)	172.83 (33.25)	174.56 (27.68)	171.87 (28.75)	0.831
Handgrip strength/(kg)	27.26 (7.48)	29.20 (7.77)	27.76 (8.39)	0.237
MVPA/(min·d^-1^)	78.96 (18.23)	90.16 (14.60)	86.21 (30.43)	0.246

Table 2. Baseline demographic and clinical characteristics of participants by exercise intensity group (N = 266). Note. ‘Mean’ represents the average, and ‘SD’ stands for standard deviation.

### Results of each variable before and after intervention

Prior to the intervention, there were no significant differences among the three groups in standing long jump, grip strength, or depressive symptoms (p > 0.05), indicating baseline comparability in these variables. Additionally, the amount of extracurricular physical activity did not differ significantly between groups (p > 0.05), as presented in Table 2. These findings confirm that any observed post-intervention differences can be attributed to the effects of the physical education programs rather than pre-existing disparities.

As summarized in Table 3, all three intensity groups showed significant improvements in standing long jump and grip strength after the 12-week teaching intervention (p < 0.05), with the vigorous-intensity group achieving the greatest gains. Improvements in depressive symptoms were significant in both the moderate- and vigorous-intensity groups (P < 0.05), with the vigorous-intensity group exhibiting the most substantial reductions.

**Table 3:** The difference of indicators among the three groups before and after 12-week physical education.

Variable		Vigorous-intensity group	Moderate-intensity group	Low-intensity group
Pre: Mean (SD)	SLJ	172.83 (33.25)	174.56 (27.68)	171.87 (28.75)
	GS	27.26 (7.48)	29.20 (7.77)	27.76 (8.39)
	DS	16.39 (9.24)	13.75 (9.96)	14.00 (12.20)
Post: Mean (SD)	SLJ	186.22 (34.41)^*^	185.90 (29.17)^*^	189.11 (31.01)^*^
	GS	29.81(8.11)^*^	31 (8.04)^*^	29.50 (7.70)^*^
	DS	10.80 (8.58)^*^	10.1 (7.94)^*^	14.34 (13.38)^2^
Post-Pre: Mean (SD)	SLJ	13.39 (10.17)	11.34 (8.41)	7.25 (11.24)^12^
	GS	2.54 (2.14)	1.80 (2.16)^1^	1.75 (2.74)^1^
	DS	−5.59 (5.44)	−3.65 (4.03)^1^	0.34 (7.25)^12^

Table 3 presents standing long jump, grip strength and depressive symptoms (mean values ± SD) at baseline and after 12 weeks in the groups, while the average exercise heart rate is between 100 and 120 (Low-intensity group), between 120 and 140 (Moderate-intensity group), and between 140 and 160 (Vigorous-intensity group). SLJ represents standing long jump (cm), GS stands for grip strength (kg), and DS refers to depressive symptoms (total CES-D score, range 0–60). The columns labeled “Pre: Mean (SD)” represent the mean and standard deviation values before the intervention, while “Post: Mean (SD)” indicate the corresponding values after the intervention. “Post-Pre: Mean (SD)” refers to the mean and standard deviation of the difference between post- and pre-intervention values. This table summarizes the observed changes across three groups following a 12-week physical education intervention at different exercise intensities. *P < 0.05 vs. baseline, ^1^P < 0.05 vs. vigorous-intensity group at the same time point, ^2^P < 0.05. vs moderate-intensity group at the same time point.

Between-group comparisons revealed that the vigorous-intensity group significantly outperformed the low-intensity group in standing long jump, grip strength, and changes in depressive symptoms (P < 0.05), the vigorous-intensity group significantly outperformed the moderate-intensity group in grip strength and depressive symptoms changes (P < 0.05), and the moderate-intensity group significantly outperformed the low-intensity group in standing long jump and depressive symptoms changes (P < 0.05). These findings suggest that higher-intensity exercise yields greater benefits for both physical and mental health outcomes.

### Multiple linear regression analysis

The multiple linear regression analysis revealed that standing long jump significantly predicted reductions in depressive symptoms across all three exercise intensity groups. Specifically, higher performance in standing long jump was associated with a greater reduction in depressive symptoms, with the strongest predictive effect observed in the vigorous-intensity group, followed by the moderate-intensity group, and lastly, the low-intensity group. The regression coefficients (B) for standing long jump were −0.16 (p = 0.03), −0.17 (p = 0.002), and −0.21 (p < 0.001) for the low-, moderate- and vigorous-intensity groups, respectively, indicating a consistent but varying degree of predictive power across intensities (Table 4).

**Table 4:** Multiple linear regression analysis of lower-limb muscle strength as a predictor of depressive symptoms.

Variable	Vigorous-intensity group			Moderate-intensity group			Low-intensity group		
	B	SE	P	B	SE	P	B	SE	P
Gender	1.46	1.18	0.22	1.77	0.90	0.05	0.87	1.52	0.57
Age	−1.81	1.18	0.13	0.22	1.43	0.88	6.27	2.16	0.01
Registered residence	−1.29	1.43	0.37	0.15	0.95	0.87	−1.99	1.76	0.26
Only child	−4.50	5.43	0.41	0.03	1.29	0.98	−11.37	5.05	0.03
Father education level	0.17	0.76	0.83	−0.25	0.51	0.63	1.08	0.71	0.13
Maternal education level	−0.46	0.73	0.53	0.45	0.46	0.33	−0.05	0.75	0.95
Family life pattern	0.58	0.92	0.53	0.05	0.65	0.94	−0.05	1.10	0.97
BMI	0.06	0.16	0.73	0.04	0.12	0.71	0.06	0.23	0.79
standing long jump	−0.21	0.06	0.00	−0.17	0.05	0.002	−0.16	0.07	0.03

Table 4 presents the results of multiple linear regression analyses investigating the relationship between changes in standing long jump and depressive symptoms across different exercise intensity groups (vigorous, moderate, and low). The dependent variable is the change in depressive symptoms before and after the intervention, while the independent variable is the change in standing long jump, controlling for potential confounders such as gender, age, registered residence, only child, parental education level, family life pattern, and BMI. All regression models utilized the following reference groups: Gender: Boy; Registered residence: Urban; Only child: Yes; Parental education level: Low; Family life pattern: Living only with parents.

Conversely, handgrip strength did not significantly predict depressive symptoms in any of the three intensity groups. The regression coefficients for handgrip strength were −0.41 (p = 0.15), −0.06 (p = 0.76), and −0.57 (p = 0.06) for the low-, moderate- and vigorous-intensity groups, respectively. These results indicate that handgrip strength was not significantly associated with depressive symptom reduction in this study (Table 5).

These findings indicate that improvements in lower-limb muscle strength play a more critical role in alleviating depressive symptoms compared to upper-limb strength, particularly at higher exercise intensities.

**Table 5:** Multiple linear regression analysis of upper-limb muscle strength as a predictor of depressive symptoms.

Variable	Vigorous-intensity group			Moderate-intensity group			Low-intensity group		
	B	SE	P	B	SE	P	B	SE	P
Gender	0.78	1.39	0.58	2.65	0.91	0.01	1.46	1.53	0.34
Age	−2.06	1.25	0.14	−0.67	1.49	0.65	4.98	2.10	0.02
Registered residence	−1.82	1.51	0.14	−0.08	1.04	0.94	−2.75	1.73	0.12
Only child	−4.19	5.88	0.24	0.62	1.36	0.65	−9.55	5.16	0.07
Father education level	−0.35	0.79	0.62	−0.18	0.55	0.74	1.07	0.72	0.14
Maternal education level	−0.14	0.77	0.86	0.54	0.49	0.27	−0.06	0.77	0.94
Family life pattern	0.60	0.98	0.55	0.08	0.70	0.91	0.12	1.15	0.92
BMI	0.16	0.17	0.36	0.04	0.13	0.75	0.12	0.23	0.61
grip strength	−0.57	0.30	0.06	−0.06	0.21	0.76	−0.41	0.28	0.15

Table 5 presents the results of multiple linear regression analyses investigating the relationship between changes in handgrip strength and depressive symptoms across different exercise intensity groups (vigorous, moderate, and low). The dependent variable is the change in depressive symptoms before and after the intervention, while the independent variable is the change in handgrip strength, controlling for potential confounders such as gender, age, registered residence, only-child status, parental education level, family life pattern, and BMI. All regression models utilized the following reference groups: Gender: Boy; Registered residence: U rban; Only child: Yes; Parental education level: Low; Family life pattern: Living only with parents.

## Discussion

This study examined the differential effects of various exercise intensities on adolescents’ muscle strength and depressive symptoms, emphasizing the critical role of exercise intensity in shaping both physical and mental health outcomes. While all exercise intensities led to improvements in muscle strength, only the moderate-intensity and vigorous-intensity programs resulted in significant reductions in depressive symptoms, with the vigorous-intensity program showing the most pronounced benefits. This underscores the capacity of higher-intensity exercise to induce neurochemical and physiological changes that positively affect mood and psychological well-being. Furthermore, improvements in lower-limb muscle strength, as assessed by the standing long jump, were identified as a key predictor of depressive symptom reduction, suggesting that targeted physical activities can play a crucial role in enhancing mental health outcomes. These findings suggest that designing physical education programs that balance both exercise intensity and psychological benefits is a strategic approach to promoting adolescent health.

### Implications for muscle strength and depressive symptoms

All three groups exhibited significant improvements in standing long jump and handgrip strength post-intervention, indicating that low-intensity exercise can also contribute to muscle strength improvements, albeit to a lesser extent compared to higher intensities. The vigorous-intensity group resulted in the greatest gains in both upper and lower limb strength, followed by the moderate-intensity group. These findings align with prior research demonstrating that higher-intensity physical activity induces greater neuromuscular adaptations more effectively than lower-intensity activity [30]. From a physiological perspective, higher load intensity during muscle contraction recruits a greater number of muscle fibers [31]. Similarly, an increased number of sets, reflecting a higher total training volume, leads to more significant muscle protein degradation. This, in turn, enhances the magnitude of supercompensation, resulting in more pronounced strength training effects [32]. Consequently, neuromuscular adaptations and strength gains are intrinsically and strongly correlated with the intensity of the training load.

Improvements in depressive symptoms were significant only in the moderate- and vigorous-intensity groups, with the vigorous-intensity group exhibiting the largest reduction in depressive scores. These improvements are clinically meaningful in the adolescent population, as even modest reductions in depressive symptoms can translate into enhanced daily functioning, better academic performance, and improved social interactions. For instance, a reduction of 3–5 points on the CES-D scale, as observed in our moderate- and vigorous-intensity groups, has been associated with decreased risk of developing major depressive disorder and improved emotional regulation in adolescents. Such changes can contribute to a lower burden of subclinical depression and may prevent the progression to more severe mental health conditions. This finding aligns with the study by Balchin et al., which examined interventions of varying intensities on patients with depression [33]. The results demonstrated that both moderate- and vigorous-intensity exercise significantly improved depressive symptoms, whereas low-intensity exercise showed no notable effect on alleviating these symptoms. The physical education instruction in this study was conducted using the Chinese Health and Physical Education Curriculum Model, which integrates diverse physical fitness activities, structured motor skill training, and instructional competitions within unpredictable teaching scenarios. This approach represents a comprehensive and multifaceted pedagogical strategy that combines various forms of instruction. Furthermore, a growing body of research indicates that moderate- to high-intensity aerobic exercise, when combined with functional training, strength training, and coordination exercises, enhances the effectiveness of depression treatment [34]. This suggests that integrated and sustained exercise interventions are significantly more effective than single-modality exercise therapies. Multi-faceted exercise regimens also better meet patients’ needs by reducing the monotony associated with single activities. They foster new perspectives and roles within group dynamics or competitive settings, promoting positive emotions, greater self-recognition, and increased trust in others [35,36].

Norris et al. found that vigorous-intensity exercise had a more pronounced effect in reducing depressive symptoms among adolescents compared to moderate-intensity exercise [37]. These findings are consistent with the results of the present study. Firstly, higher-intensity exercises typically induce greater neurochemical changes, which are crucial for mood regulation and alleviating depressive symptoms [22,24,38]. Furthermore, the psychological benefits of vigorous-intensity exercise, such as increased self-esteem and mood elevation, are often more marked, potentially due to the higher physical exertion and the associated sense of accomplishment. Consequently, although moderate-intensity exercise also contributes to mental health improvements, vigorous-intensity exercise appears to exert a more robust influence on reducing depressive symptoms.

### Predictive role of muscle strength in depressive symptoms

The association between lower-limb muscle strength and reductions in depressive symptoms underscores the importance of incorporating targeted strength-training exercises into physical education curricula. These findings align with existing evidence, which demonstrates that muscle strength, particularly in the lower limbs, is inversely associated with depressive symptoms [27,39,40]. In contrast, handgrip strength was not found to significantly predict reductions in depressive symptoms, suggesting that specific muscle groups may have distinct roles in psychological outcomes. Future studies could further investigate the underlying mechanisms that link lower-limb strength to improved mental health, such as increased physical self-efficacy or enhanced neurochemical responses [26,40].

The relationship between lower limb muscle strength and the alleviation of depressive symptoms could be underpinned by neurobiological mechanisms. Exercise, particularly at moderate to vigorous intensities, has been shown to elevate BDNF levels, which play a critical role in neuroplasticity and mood regulation [24]. This aligns with findings that higher levels of BDNF are associated with improved depressive symptoms following physical activity interventions. Furthermore, neuroendocrine adaptations to exercise, such as the regulation of the hypothalamic-pituitary-adrenal (HPA) axis, may contribute to the psychological benefits observed. Exercise-induced reductions in cortisol levels have been linked to lower depressive symptom severity [41]. These mechanisms might explain the stronger association between lower limb strength and depressive symptom improvement, given the greater systemic engagement and metabolic demand of exercises targeting larger muscle groups [40].

The lack of a significant predictive relationship between handgrip strength improvements and alleviation of depressive symptoms may be attributed to several factors. Firstly, handgrip strength predominantly reflects localized upper-limb muscle performance, which might engage systemic physiological and neurobiological mechanisms less robustly compared to lower-limb or full-body exercises [42]. For instance, lower-limb activities, such as jumping, elicit greater cardiovascular and metabolic responses, potentially driving more pronounced neurochemical changes beneficial for mood regulation [43]. Secondly, handgrip strength may lack the functional and psychological relevance necessary to exert a significant influence on depressive symptoms. Unlike whole-body movements, which can enhance physical self-efficacy and social interaction, handgrip strength improvements may not yield substantial psychological or social reinforcement [44]. Finally, a potential ceiling effect in handgrip strength among adolescents could reduce its variability, thereby weakening its statistical association with depressive outcomes. Future studies should investigate the role of various muscle groups and exercise modalities to further elucidate these relationships.

### Practical recommendations

Schools should integrate structured physical education programs with tailored intensity levels into their curricula to enhance adolescents’ physical and mental health. Vigorous intensity exercises are recommended for their pronounced benefits in improving muscle strength and reducing depressive symptoms, particularly for students at higher mental health risk. However, individualized assessment and professional guidance are advised when implementing high-intensity programs for vulnerable populations. Moderate-intensity programs may offer a more accessible and sustainable option for broader student participation. Furthermore, regular assessments of functional muscle strength, such as standing long jump performance, should be implemented to monitor both physical and psychological progress, facilitating early identification of students who may benefit from targeted interventions.

### Limitations and future research

Despite the promising findings, this study has several limitations. First, the reliance on self-reported measures for depressive symptoms introduces the possibility of response biases, which may affect the accuracy of the findings. Future studies should incorporate objective measures of mental health, such as neuroimaging or biochemical markers, to provide a more comprehensive understanding of the psychological outcomes associated with physical activity. Second, the sample was limited to eighth-grade students from a specific geographic region, which may restrict the generalizability of the results. Future investigations should include diverse age groups, socio-economic backgrounds, and cultural contexts to validate the findings across broader populations. Third, Although the use of Polar OH1 sensors ensured accurate heart rate monitoring and adherence to prescribed exercise intensities, the associated cost may limit the widespread application of such technology in resource-constrained educational settings. Future implementations could explore more affordable alternatives, such as validated consumer-grade wearables or group-based pulse monitoring techniques, to balance accuracy with practicality.

## Conclusion

In conclusion, this study demonstrates that all three exercise intensity levels can improve muscle strength, with the most significant gains observed in vigorous intensity programs. However, only moderate and vigorous intensity exercises were effective in alleviating depressive symptoms, with the latter yielding the most pronounced improvements. Furthermore, the increase in lower-limb muscle strength significantly predicted the reduction in depressive symptoms, underscoring its critical role in mental health enhancement. These findings highlight the need for school-based physical education programs to incorporate targeted, intensity-appropriate exercises that optimize both physical and psychological outcomes in adolescents.

## Supporting information

S1 Appendix CONSORT checklist. Completed CONSORT checklist for randomized controlled trials.
(DOCX)

S2 Appendix Human participants research checklist. Checklist confirming compliance with ethical standards for human participants research.
(DOCX)

S1 Protocol Original research plan. Detailed research protocol in Chinese as submitted for ethical approval.
(DOCX)

S2 Protocol Research plan (English version). English translation of the original research plan.
(DOCX)

S1 Fig Experimental photographs. The ActiGraph device used for measuring physical activity.
(JPG)

S2 Fig Experimental photographs. Measurement of participants’ height and weight.
(JPG)

S3 Fig Experimental photographs. Motor skill exercises conducted in the physical education classroom.
(JPG)

S4 Fig Experimental photographs. Measurement of handgrip strength.
(JPG)

S5 Fig Experimental photographs. Heart rate monitoring during the physical education class.
(JPG)

S6 Fig Experimental photographs. Information collected through a questionnaire.
(JPG)

S7 Fig Experimental photographs. Physical fitness training in the physical education class.
(JPG)

S8 Fig Experimental photographs. Measurement of standing long jump.
(JPG)

S1 Dataset Research data. Anonymized dataset used for statistical analysis in this study.
(XLS)
