# Supplementary material for: Effects of varying exercise intensities on muscle strength and depressive symptoms in Chinese adolescents: A 12-week randomized controlled trial
Source: PLoS One. 2025 Nov 21;20(11):e0336894. doi: 10.1371/journal.pone.0336894 (PMC12637978; doi:10.1371/journal.pone.0336894)
Supplement: S1 Protocol — Detailed research protocol in Chinese as submitted for ethical approval. (DOCX) [file pone.0336894.s003.docx]

**研究方案**

**一、课题基本信息**

**方案名称：**基于代谢组学探讨中国健康体育课程模式对学生身心健康的影响效果及机制

**主要研究者：**常青

**二、研究方案**

1.研究背景

2021年教育部公布的第八次全国学生体质与健康调研结果显示，2019年全国6~22岁学生体质健康达标优良率仅为23.8%。体育课是学生健康促进的主要渠道，长期以来我国传统体育教学对学生总体健康水平影响有限。为解决学生体质健康水平近30年来持续下降、肥胖率居高不下以及体育活动和学习生活中意志薄弱、活力不够、缺少交往、性格孤僻、焦虑抑郁等一系列身心健康问题，我国体育与健康课程标准研制组和修订组组长季浏教授，带领其团队经过长期的理论研究和实践探索构建了中国健康体育课程模式。中国健康体育课程模式是我国迄今为止第一个在期刊上公开发表的完全符合我国《体育与健康课程标准》要求的体育课程模式，自提出以来通过建立实验基地学校以及推广传播，越来越多的教师尝试采用该模式进行教学，越来越多的学生从中受益。

代谢组学是继基因组学和蛋白质组学之后发展起来的一门学科，其可在不设定将要检测的具体指标时对代谢物（分子量小于1000）“全景式”扫描，再对检测的显著代谢标志物和差异物进行分析，观察机体整体的代谢情况。代谢物是上游生化过程的最终终点，并密切反映所表达的表型，而运动往往会伴随着机体的糖代谢 、氨基酸代谢、脂质代谢等发生变化，机体内小分子物质也会发生质或量的改变，这些代谢物的变化可以为运动处方选择合适的运动强度提供足够的理论依据。基于质谱的大通量代谢组学数据有助于机体整体代谢网络的构建和代谢靶标的发掘。质谱技术的进步促进了越来越多关于急性或慢性运动负荷对全身代谢物反应的研究。尿液是机体新陈代谢的重要媒介，也是代谢产物的主要载体，并且尿液代谢组学样本获取时对受试者无创伤，优势明显。

人体是一个系统的有机体，运动训练作为对机体的一种干预，可对机体产生广泛而深远的影响，借助代谢组学的思路和技术手段，找出运动干预前后机体的差异代谢物，并对其涉及的代谢通路进行分析，观察受试者在训练期间代谢物的

变化，这将为研究运动促进健康的内在分子机制、科学制定运动处方提供依据。但目前我国对于运动代谢组学的研究尚处于起步阶段，代谢组学在学校体育的应用更是鲜有报道。

既往研究已表明，中国健康体育课程模式下的体育教学可有效提高学生身心健康水平，且效果显著优于传统体育教学。但是，关于中国健康体育课程模式促进学生身心健康的内在机制方面的研究却未见报道。本研究运用中国健康体育课程模式进行12周体育课堂教学，在教学前后测试相关身体健康和心理健康指标，通过非靶向代谢组学技术检测教学前后代谢物的变化，分析中国健康体育课程模式1次性教学和长期教学对学生身心健康水平的改善效果及其产生机制。旨在为中国健康体育课模式提高学生身心健康提供新思路。

2.研究目的

本研究运用中国健康体育课程模式进行12周体育课堂教学，在教学前、后测试相关体质健康和心理健康指标，通过非靶向代谢组学技术检测教学前、后代谢物的变化，分析1次性体育教学和长期体育教学对学生身心健康水平的改善效果及其代谢机制，旨在为中国健康体育课模式提高学生身心健康提供新思路。

3.研究方法

3.1实验对象与分组

采用G*power软件进行样本量计算，选择“t tests”中的“Means: Difference between two independent means (two groups)”，假设中等效应量为0.5，α err prob为0.05，Power为0.80，两组样本量相等，计算出研究的最小样本量为64。

本研究为随机对照试验，随机抽取6个相同年级的班级，3个为实验班，另3个为对照班。研究者事先将每个受试班级的分配信息（例如，实验组或对照组）写在纸条上，并将这些纸条放入信封中。每个信封内含有该受试班级的随机分配信息。分别对两组受试者及其家长进行实验内容及实验过程中注意事项的介绍，让其对本次实验有详细了解，根据实验纳入标准及排除标准，征求学生受试者及家长意见后填写知情同意书。

3.2受试者入选标准、排除标准、剔除标准、中途退出标准

纳入标准：①中小学在校学生；②能够完成12周的干预及干预前、第1次干预后、干预12周后的三次测试者。

排除标准：①有体育运动禁忌症者；②严重心脏或肌骨疾病者；③近期生病、外伤、慢性病、服药或处于生理期（女生）的学生；④未签署知情同意书者。

剔除标准：①缺勤三分之一以上者；②因家庭或个人原因无法完成测试者；③干预过程中受试者参加了其它体育运动培训班违背干预方案者。

中途退出标准：转学或其它原因未能继续参加研究者。

3.3实验内容

本研究主要在中小学内开展，需要6个班级（约300名学生）自愿参加。

3个作为实验班，采用中健康体育课程模式进行体育教学；3个作为对照班，采用正常传统体育教学。

实验班运用中国健康体育课程模式对学生进行 12 周体育教学，对照班采用正常传统体育教学方式进行 12 周体育教学。在教学前、后对学生身心健康指标进行测试。其中，体质健康指标按照《国家学生体质健康标准（2014 年修订）》进行测试，包括身体形态、肺活量、速度、力量、柔韧、灵敏等指标；心理健康指标采用问卷调查进行测试，包括健康行为、体育品德、抑郁症状等指标；随机选取40名学生（实验组20名，对照组20名）在教学前、第1次教学后、12周教学后分别采集15ml尿液，采用非靶向代谢组学技术检测体内代谢物的变化，探讨运动促进健康的内在机制。

另外，教学期间两组学生均要佩戴 polor 心率仪监测运动心率，运用量表检测课外运动量、饮食和睡眠等，排除其它指标产生的代谢干扰。

3.4干预方案

实验班和对照班均为每节课40min，每周3节课，共36次课，运动技能均为排球运动。

中国健康体育课程模式教学时间分配为：准备部分为7min（课堂常规1min，准备活动6min），基本部分为30min（运动技能20min，体能10min），结束部分为3min（放松活动2min，课堂小结1min），中国健康体育课程模式教学方法如表1所示。

传统体育教学模式的教学时间分配为：准备部分为10min（课堂常规3min，准备活动7min），基本部分为30min（单一动作技术的多方法练习，无比赛），结束部分为5min（放松活动3min，课堂小结2min）。主要特征是（1）运动负荷小，达不到中国健康体育课程模式的要求；（2）无专门的体能练习；（3）单一动作技术的教学。

表1.中国健康体育课程模式教学要求

| 中国健康体育课程模式 | |
| --- | --- |
| 运动负荷 | 75%以上的运动密度，140-160次/分的运动强度 |
| 体能练习 | 每节体育课要有10分钟的体能练习，体能练习应注重多样化、趣味性和“补偿性” |
| 运动技能 | 时间保证在20 min左右，既要有单个技术和组合技术的学练，更要把单个技术和组合技术的学练与完整活动或比赛有机联系，重视在运用中掌握和提高技术 |
| 学习目标 | 提高学生的体质健康水平和心理健康水平 |
| 教学内容 | 受学生喜爱、有助于促进学生身心健康的内容 |
| 教学方式 | 从以教为主向以学为主转变, 关键是提倡多样化的教学方式 |
| 课堂氛围 | 师生和谐互动、情绪饱满高昂、场景活泼热烈、气氛积极向上 |
| 总体评价 | 过程性和结果性评价相结合 |

3.5实验步骤

告知研究过程及注意事项——根据班级分组——教学前测试（包括身体健康指标、心理健康指标测试以及代谢物采集）——第1次教学后检测（仅代谢物采集）——进行12周体育教学（实验班采用中国健康体育课程模式，对照班采用传统体育教学）——教学后测试（与教学前测试一致）。

3.6测试指标及方法

体质健康指标按照《国家学生体质健康标准（2014 年修订）》进行测试，包括身体形态、心肺耐力、速度、力量、柔韧、力量耐力等指标。体成分指标采用身体质量指数和腰围；心肺耐力指标采用肺活量；力量指标采用1min仰卧起做和立定跳远；速度指标采用50m跑；柔韧性指标采用坐位体前屈；力量耐力指标采用1000m或800m跑。主要测试方法如下：

①身体质量指数：受试者赤脚站在身高计踏板上，背靠立柱、两脚跟并拢，身体直立，受试者的脚跟、骶骨和肩胛部三部分紧靠立柱，头部保持正直（即两眼眶下缘和耳屏上沿呈一条水平线），记录压板对应的数值。身高数值的记录以厘米（cm）为单位，精确到小数点后1位。体重以（kg）为单位，精确到小数点后1位。按公式得出受试者的身体质量指数：BMI=weigh/heigh2(kg/m2)。

②肺活量：使用干燥的一次性口嘴（非一次性口嘴，则每换测试对象需消毒一次，每测一人时将口嘴下倒出唾液并注意消毒后必须使其干燥）。以中等速度和力度尽全力吹气效果最好。令受试者手持吹气口嘴，测试时，受试者进行一两次较平日深一些的呼吸动作后，更深地吸一口气，屏住气向口嘴处慢慢呼出至不能再呼为止，防止此时从口嘴处吸气，测试中不得中途二次吸气。吹气完毕后，液晶屏上最终显示的数字即为肺活量毫升值。选取最大值作为测试结果。以毫升为单位，不保留小数。

③1min仰卧起坐（女）/引体向上（男）：1min仰卧起坐的测试为，测试者平躺于垫子上，两臂交叉置于胸前，手掌心贴紧两肩，双脚平放在地面，膝关节呈90°。开始测试时，测试者上体前倾至两肘关节触及或超过膝关节为完成1次。最终记录测试者在30 s内完成次数。引体向上的测试为受试者跳起双手正握杠，两手与肩同宽成直臂悬垂，双手正握单杠，待身体静止后，两臂同时用力引体（身体不能有附加动作），上拉到下颌超过横杠上缘为完成一次，记录引体次数。

④立定跳远：受试者两脚自然分开站立, 站在仪器起跳线后, 脚尖不得踩线, 不得有垫步或连跳动作。起跳线至最近着落点后缘的垂直距离为测量值, 脚跟落点必须完全进入测试区。测量3次，取最大记录值。

⑤50m跑：在平坦地面上画长50米的直线跑道，跑道线要清晰， 用电子表进行计时, 一道一表。在起跑道上, 发出“预备”的口令后吹哨, 此时受试者开跑, 同时测试者开始计时, 以受试者到达终点结束计时, 记录成绩。

⑥坐位体前屈：选用日本坐位体前屈仪器进行测试。准备动作时，测试者坐于平坦地面或垫子上，臀部、背部、肩部和脑后部位贴紧墙壁，双臂伸直，掌心朝下双手置于测试仪器板面，两腿伸直。开始测试时，上体前屈推动测试仪器缓缓向前，直至不能往前推动测试仪器为止，记录成绩以cm为单位。

⑦800m跑（女）/1000m跑（男）：测试安排在400米的田径跑道进行，受试者站立式起跑。当听到“鸣枪”之后开始起跑，当受试者的躯干部到达终点线垂直面时停表。以分、秒为单位记录测试成绩，不计小数。

3.7.心理指标的检测

健康行为和体育品德的测试采用戴圣婷博士的《小学生体育与健康学科核心素养评价量表》；抑郁症状采用中文版《流调中心抑郁量表》。

3.8.代谢组学方法

随机抽取40名学生（实验班20名，对照班20名），分别在教学前、第1次教学后和12周教学后采集15ml尿液样本，通过处理后基于液相色谱联合质谱法的非靶向代谢组学分析1次性教学前后和长期教学前后差异代谢物，并进行相关代谢通路分析，获得干预前后的代谢指纹谱。通过主成分分析、正交偏最小二乘法-判别分析明确干预前后代谢谱之间的差异，进一步通过组内代谢物定量均值的t检验、OPLS-DA和聚类分析筛选差异代谢物。将代谢物的质荷比与人类代谢组数据库及KEGG数据库进行匹对和分析对差异代谢物进行结构鉴定、富集及拓扑分析，探讨中国健康体育课程模式1次性教学前后和长期教学前后的差异代谢物涉及的代谢通路。

3.9课外运动量、饮食和睡眠

课外运动量的监测采用《儿童青少年身体活动调查问卷(PAQ)》；睡眠监测采用《匹茨堡睡眠量表》；饮食的监测采用《饮食行为量表》。

3.10.数据统计与分析

本研究采用 SPSS 26. 0 版统计软件对实验数据进行归纳分析，采用Shapiro -Wilk 法行正态性检验，数据采用平均值±标准差形式表示。采用配对样本T检验比较干预前后各指标差异；采用独立样本T检验比较干预前两组受试者组间基础指标以及两组干预前后各指标的差值差异。P<0.05 为有统计学差异。

1. 预期的进度和完成日期

4.1准备阶段（2024年5月~2024年8月）

（1）寻找实验基地学校，与相关体育教师进行交流和培训。

（2）学习相关理论知识，制定实验方案，完成前期的资料汇总。

4.2实施阶段（2024年09月~2025年01月）

（1）收集前测数据，测试实验前各体质健康和心理健康指标及采集尿液制作样本进行实验干预。

（2）收集后测数据，测试实验后各体质健康指标及采集尿液制作样本。

4.3结题阶段（2025年01月~2025年06月）

（1）整理课题材料，进行总结、评估，完成结果分析。

（2）撰写相关论文及结题报告，申报并迎接评审。
