# Supplementary material for: Effects of varying exercise intensities on muscle strength and depressive symptoms in Chinese adolescents: A 12-week randomized controlled trial
Source: PLoS One. 2025 Nov 21;20(11):e0336894. doi: 10.1371/journal.pone.0336894 (PMC12637978; doi:10.1371/journal.pone.0336894)
Supplement: S2 Protocol — English translation of the original research plan. (DOCX) [file pone.0336894.s004.docx]

**Research Proposal**

**Basic Information of the Project**

Project Title: Exploring the Impact and Mechanisms of the Health and Physical Education Curriculum Model of China on Students' Physical and Mental Health Based on Metabolomics

Principal Investigator: Qing Chang

**Research Plan**

**1. Background**

According to the results of the 8th National Physical Fitness and Health Survey published by the Ministry of Education in 2021, the rate of students aged 6 to 22 years meeting physical health standards in 2019 was only 23.8%. Physical education classes are the primary channel for promoting student health. However, traditional physical education teaching in China has had limited impact on the overall health level of students. To address the continuous decline in students' physical health over the past 30 years, the high obesity rate, and a series of physical and mental health issues such as weak willpower, lack of vitality, social withdrawal, introversion, anxiety, and depression in physical activities and daily life, Professor Ji Liu, the head of the Sports and Health Curriculum Standard Development and Revision Team, along with his research team, conducted long-term theoretical research and practical exploration to create the China Health and Physical Education Curriculum Model. This model, which is the first publicly published physical education curriculum model in China that fully aligns with the "Physical Education and Health Curriculum Standards," has been increasingly adopted by teachers and has benefited more and more students through the establishment of experimental base schools and its widespread promotion.

Metabolomics, a discipline developed after genomics and proteomics, enables a "holistic" scan of metabolites (molecules smaller than 1000 Da) without predefined target indicators, followed by analysis of significant metabolic biomarkers and differences, to observe the overall metabolic status of the body. Metabolites are the final endpoints of upstream biochemical processes and closely reflect the expressed phenotype. Exercise often induces changes in body metabolism, such as glucose metabolism, amino acid metabolism, and lipid metabolism, leading to alterations in the quantity or quality of small molecules in the body. These changes in metabolites provide a theoretical basis for selecting appropriate exercise intensity in exercise prescriptions. High-throughput metabolomics data based on mass spectrometry are helpful for constructing the body's overall metabolic network and discovering metabolic targets. Advances in mass spectrometry technology have facilitated research into the body's response to acute or chronic exercise loads at the metabolic level. Urine, an important medium for metabolism in the body, is the main carrier of metabolic products and has the advantage of being non-invasive when obtaining metabolomic samples, offering clear advantages.

The human body is an organic system, and exercise training, as an intervention, can have broad and profound effects on the body. By utilizing metabolomics concepts and techniques, it is possible to identify differential metabolites before and after exercise intervention and analyze the metabolic pathways involved. This allows for the observation of changes in metabolites during training, providing insights into the molecular mechanisms through which exercise promotes health and supporting the scientific formulation of exercise prescriptions. However, research on exercise metabolomics in China is still in its early stages, with very few reports on the application of metabolomics in school sports.

Previous studies have shown that physical education teaching under the China Health and Physical Education Curriculum Model can effectively improve students' physical and mental health, with results significantly better than traditional physical education teaching. However, there have been no reports on the internal mechanisms of the China Health and Physical Education Curriculum Model in promoting students' physical and mental health. This study will employ the China Health and Physical Education Curriculum Model for 12 weeks of physical education instruction. Before and after the teaching, relevant physical and psychological health indicators will be tested. Using non-targeted metabolomics technology, changes in metabolites before and after teaching will be detected, and the improvement effects and mechanisms of both one-time and long-term teaching using this model on students' physical and mental health will be analyzed. The goal is to provide new insights into improving students' physical and mental health through the China Health and Physical Education Curriculum Model.

**2. Research Objective**

This study will use the China Health and Physical Education Curriculum Model for 12 weeks of physical education teaching. Relevant physical health and psychological health indicators will be tested before and after the teaching. Changes in metabolites before and after the teaching will be detected through non-targeted metabolomics technology. The study will analyze the effects and metabolic mechanisms of one-time and long-term teaching using the China Health and Physical Education Curriculum Model on improving students' physical and mental health levels, aiming to provide new insights into improving students' physical and mental health through this model.

1. **Research Methodology**

- 1. Participants and Grouping

The sample size was calculated using G*Power software, selecting “Means: Difference between two independent means (two groups)” under “t tests.” The assumptions included a medium effect size of 0.5, an α error probability of 0.05, and a power (1 - β error probability) of 0.80, with equal sample sizes in both groups. The minimum required sample size for the study was calculated to be 64.

This study employed a randomized controlled trial design. Six classes of the same grade were randomly selected, with three assigned to the experimental group and the other three to the control group. The researchers wrote the allocation information for each participating class (e.g., experimental or control group) on slips of paper and placed these slips into envelopes. Each envelope contained the random allocation information for the respective class. Both the participants and their parents in the two groups were introduced to the content of the experiment and the precautions during the experimental process to ensure they had a detailed understanding. After obtaining the opinions of the student participants and their parents based on the inclusion and exclusion criteria, informed consent forms were signed.

3.2 Inclusion Criteria, Exclusion Criteria, Criteria for Removal, and Mid-study Withdrawal Criteria

Inclusion criteria: (1) Primary and secondary school students; (2) Able to complete the 12-week intervention and the three tests before the intervention, after the first intervention, and after 12 weeks of intervention.

Exclusion criteria: (1) Students with contraindications to physical activity; (2) Students with severe cardiovascular or musculoskeletal diseases; (3) Students who are sick, injured, have chronic diseases, are taking medication, or are in their menstrual period (female students); (4) Students who have not signed the informed consent form.

Criteria for removal: (1) Students absent for more than one-third of the sessions; (2) Students who are unable to complete the tests due to personal or family reasons; (3) Students who participated in other sports training sessions during the intervention, violating the intervention plan.

Mid-study withdrawal criteria: Students who transferred schools or could not continue participating in the study due to other reasons.

3.3Experiment Content

This study was primarily conducted in primary and secondary schools, involving six classes (approximately 300 students) who voluntarily participated. Three classes were designated as experimental groups, which adopted the Healthy Physical Education Curriculum Model of China for physical education instruction, while the other three classes served as control groups, receiving traditional physical education instruction.

The experimental groups underwent 12 weeks of physical education instruction based on the Healthy Physical Education Curriculum Model of China, whereas the control groups followed the conventional traditional physical education instruction for the same duration. Pre- and post-instruction assessments were conducted to measure the students' physical and mental health indicators. The physical health indicators were evaluated according to the National Student Physical Health Standard (Revised in 2014), which included anthropometric measurements, vital capacity, speed, strength, flexibility, agility, and other indicators. The mental health indicators were assessed through questionnaires, covering healthy behaviors, sportsmanship, depressive symptoms, and other indicators. Additionally, 40 students (20 from the experimental group and 20 from the control group) were randomly selected to provide 15 ml of urine samples before instruction, after the first session of instruction, and after 12 weeks of instruction. Non-targeted metabolomics techniques were employed to detect changes in metabolites within the body, in order to explore the underlying mechanisms by which exercise promotes health.

Furthermore, during the instructional period, all students in both groups were required to wear Polar heart rate monitors to track their exercise heart rates. Questionnaires were also used to assess extracurricular physical activity, diet, and sleep patterns, in order to eliminate metabolic interferences resulting from other factors.

3.4 Intervention Program

Both the experimental and control groups had 40-minute lessons per session, three times a week, for a total of 36 lessons. The sport practiced was volleyball.

The time allocation for the Chinese Health Physical Education curriculum model was as follows: 7 minutes for the preparation phase (1 minute for class routines, 6 minutes for warm-up activities), 30 minutes for the main phase (20 minutes for skill training, 10 minutes for physical fitness exercises), and 3 minutes for the conclusion phase (2 minutes for relaxation, 1 minute for class summary). The teaching methods for the Chinese Health Physical Education curriculum model are shown in Table 1.

**Table 1. Teaching Requirements of the Health Physical Education Curriculum Model of China**

| ****The Health Physical Education Curriculum Model of China**** | |
| --- | --- |
| **Exercise Load** | Exercise density above 75%, intensity of 140-160 beats/min |
| **Physical Fitness Exercises** | Each lesson includes 10 minutes of physical fitness exercises, which should be varied, engaging, and "compensatory" |
| **Sport Skills** | Time should be around 20 minutes, focusing on individual techniques, combined techniques, and integrating both into complete activities or competitions. Emphasis on mastering and improving techniques through application |
| **Learning Objectives** | Improve students' physical health and mental health levels |
| **Teaching Content** | Content that students enjoy and which contributes to their physical and mental health |
| **Teaching Method** | Shift from teacher-centered to student-centered, with an emphasis on diversified teaching methods |
| **Classroom Atmosphere** | Harmonious teacher-student interaction, high energy, lively scenes, and a positive atmosphere |
| **Overall Evaluation** | Combination of process-oriented and result-oriented evaluations |

3.5 Experimental Procedure

Informing the research process and precautions—Grouping by class—Pre-instruction testing (including physical health indicators, mental health indicators, and metabolite collection)—Testing after the first session of instruction (metabolite collection only)—Conducting 12 weeks of physical education instruction (experimental groups adopting the Chinese Healthy Physical Education Curriculum Model, control groups adopting traditional physical education instruction)—Post-instruction testing (consistent with pre-instruction testing).

3.6 Test Indicators and Methods

The physical health indicators were tested according to the "National Physical Fitness Standards for Students (2014 Revision)," including body shape, cardiorespiratory endurance, speed, strength, flexibility, and muscular endurance. The body composition indicators were assessed using body mass index (BMI) and waist circumference. Cardiorespiratory endurance was tested using vital capacity. Strength was measured by a 1-minute sit-up test (female) or pull-up test (male). Speed was measured using the 50-meter run. Flexibility was tested using the sit-and-reach test. Muscular endurance was measured with an 800-meter run (female) or 1000-meter run (male). The main testing methods are as follows:

1) Body Mass Index (BMI): The participant stands barefoot on a height measuring platform with their back against the column, feet together, and body straight. The participant's heels, sacrum, and scapula should be in contact with the column, with the head aligned horizontally. The height is recorded in centimeters (cm), accurate to one decimal place. Weight is recorded in kilograms (kg), also accurate to one decimal place. BMI is calculated using the formula: BMI = weight / height² (kg/m²).

2) Vital Capacity: A dry, disposable mouthpiece (or disinfected non-disposable mouthpiece) is used. The participant should take a deep breath and exhale into the mouthpiece until no more air can be expelled, avoiding inhalation during the test. The final reading on the LCD screen is recorded as the vital capacity in milliliters (mL), and the maximum value is selected as the result.

3) 1-Minute Sit-Up Test (Female) / Pull-Up Test (Male): For sit-ups, the participant lies on a mat with arms crossed over their chest, palms pressed against the shoulders, and feet flat on the ground, with knees at a 90° angle. Each sit-up is counted when the elbows touch or exceed the knees. The number of sit-ups performed in 30 seconds is recorded. For pull-ups, the participant grasps a horizontal bar with both hands shoulder-width apart, hanging with straight arms. After stabilizing their body, they pull up until the chin is above the bar, and the number of successful pull-ups is recorded.

4) Standing Long Jump: The participant stands with feet apart behind the take-off line, ensuring no part of the foot crosses the line. The distance from the take-off line to the nearest point of landing is measured, and the heel must land fully within the test area. The best of three jumps is recorded.

5) 50-Meter Sprint: A 50-meter straight track is marked on flat ground, and the time is recorded with a stopwatch. The test starts with the "Ready" command, and the participant begins running when the whistle is blown. Timing ends when the participant reaches the finish line.

6) Sit-and-Reach Test: The participant sits on the floor or mat with their back, hips, and shoulders against the wall, legs straight, and arms extended forward with palms facing down. The participant pushes the testing device forward as far as possible without bouncing, and the result is recorded in centimeters (cm).

7) 800-Meter Run (Female) / 1000-Meter Run (Male): The test is conducted on a 400-meter athletic track. The participant starts from a standing position, and timing starts upon hearing the starting gun and ends when the torso crosses the finish line. The result is recorded in minutes and seconds without decimals.

3.7 Psychological Indicators

Health behaviors and sports ethics are assessed using Dr. Shengting Dai's "Core Competency Evaluation Scale for Primary School Physical Education and Health." Depression symptoms are measured using the "Depression Scale from the Center for Disease Control."

3.8 Metabolomics Method

Forty students were randomly selected (20 from the experimental group and 20 from the control group). Urine samples (15 ml) were collected from these students before instruction, after the first session of instruction, and after 12 weeks of instruction. The samples were processed and analyzed using non-targeted metabolomics based on liquid chromatography–mass spectrometry (LC-MS) to identify differential metabolites before and after a single session of instruction, as well as before and after long-term instruction. Metabolic pathway analysis was also conducted to obtain metabolic fingerprints before and after the intervention.

Principal component analysis (PCA) and orthogonal partial least squares discriminant analysis (OPLS-DA) were employed to elucidate the differences in metabolic profiles before and after the intervention. Differential metabolites were further screened using t-tests of intragroup mean metabolite concentrations, OPLS-DA, and clustering analysis. The mass-to-charge ratios (m/z) of the metabolites were matched and analyzed against the Human Metabolome Database and the Kyoto Encyclopedia of Genes and Genomes (KEGG) database for structural identification, enrichment, and topological analysis of the differential metabolites. This approach was used to explore the metabolic pathways involved in the differential metabolites before and after a single session of instruction, as well as before and after long-term instruction, under the Healthy Physical Education Curriculum Model of China.

3.9 Extracurricular Physical Activity, Diet, and Sleep

Extracurricular physical activity is monitored using the "Physical Activity Questionnaire for Children and Adolescents (PAQ)." Sleep is assessed using the "Pittsburgh Sleep Quality Index." Diet is monitored using the "Dietary Behavior Scale."

3.10 Data Statistics and Analysis

Data will be analyzed using SPSS 26.0. Normality tests will be performed using the Shapiro-Wilk method, and data will be presented as mean ± standard deviation. Paired sample t-tests will be used to compare differences before and after intervention for each indicator. Independent sample t-tests will be used to compare baseline indicators between the two groups and differences between the pre- and post-intervention values. Statistical significance is set at P<0.05.

1. **Expected Timeline and Completion Dates**

4.1 Preparation Phase (May 2024–August 2024)

(1) Identify experimental base schools and engage in communication and training with relevant physical education teachers.

(2) Acquire relevant theoretical knowledge, develop the experimental plan, and complete the preliminary collection and summarization of materials.

4.2 Implementation Phase (September 2024–January 2025)

(1) Collect pre-intervention data, including testing physical and mental health indicators and collecting urine samples for the experimental intervention.

(2) Collect post-intervention data, including testing physical health indicators and collecting urine samples after the intervention.

4.3 Conclusion Phase (January 2025–June 2025)

(1) Organize research materials, conduct summaries and evaluations, and complete the analysis of results.

(2) Write relevant papers and the final project report, and prepare for and undergo the evaluation process.
