# Supplementary figures and images for: Effects of varying exercise intensities on muscle strength and depressive symptoms in Chinese adolescents: A 12-week randomized controlled trial
Source: PLoS One. 2025 Nov 21;20(11):e0336894. doi: 10.1371/journal.pone.0336894 (PMC12637978; doi:10.1371/journal.pone.0336894)

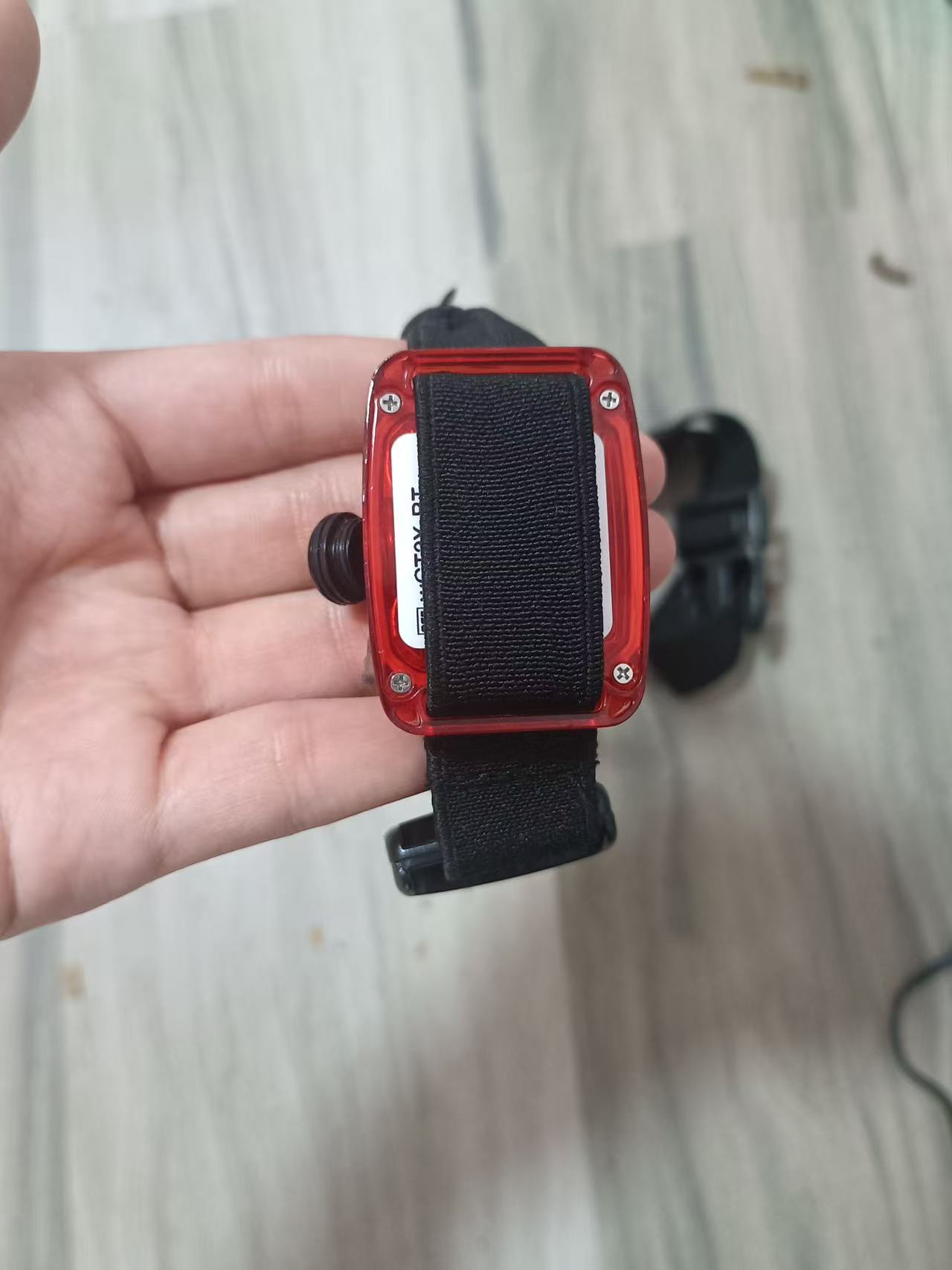

Supplement: S1 Fig — The ActiGraph device used for measuring physical activity. (JPG) [file pone.0336894.s005.jpg]

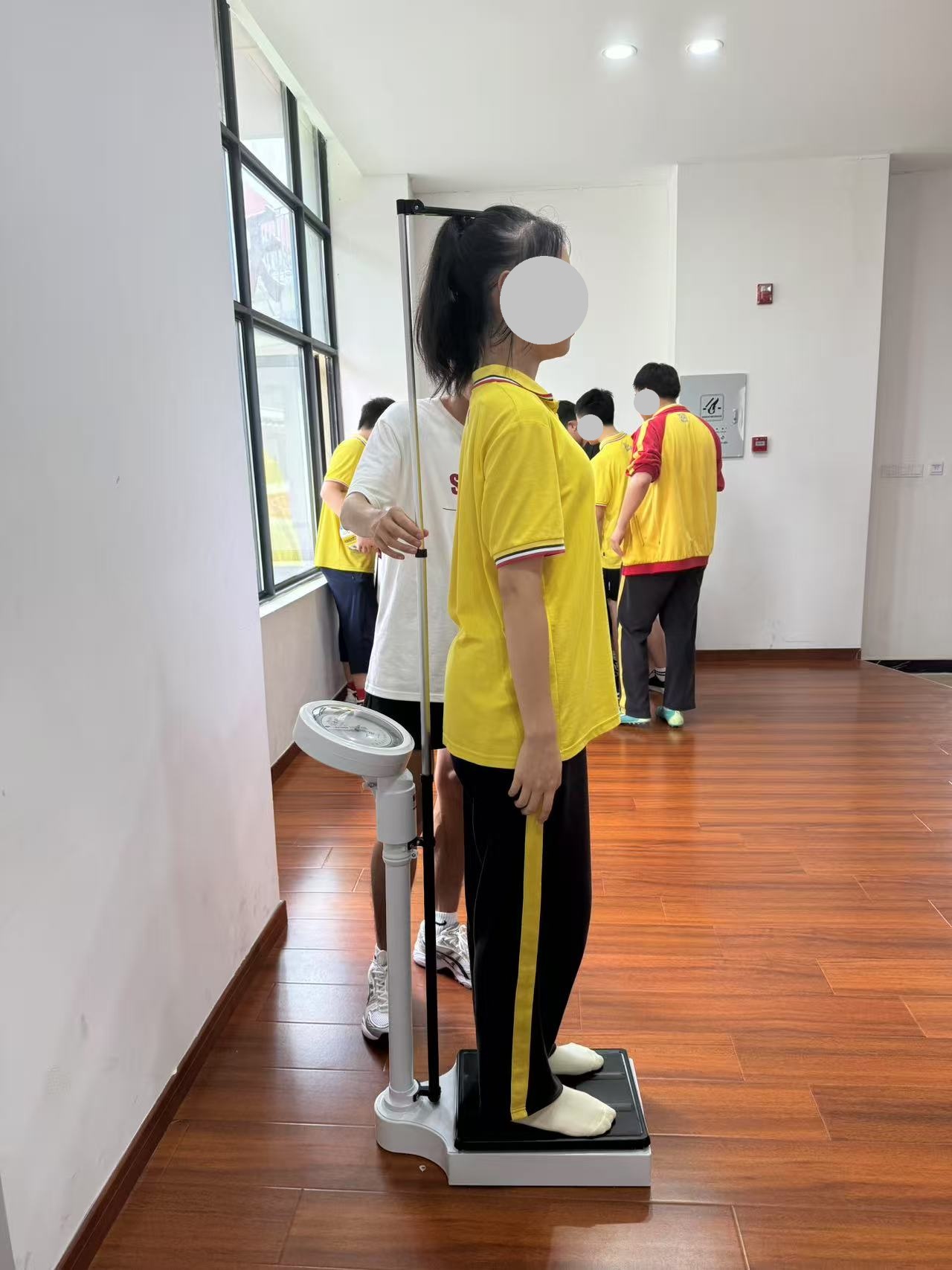

Supplement: S2 Fig — Measurement of participants’ height and weight. (JPG) [file pone.0336894.s006.jpg]

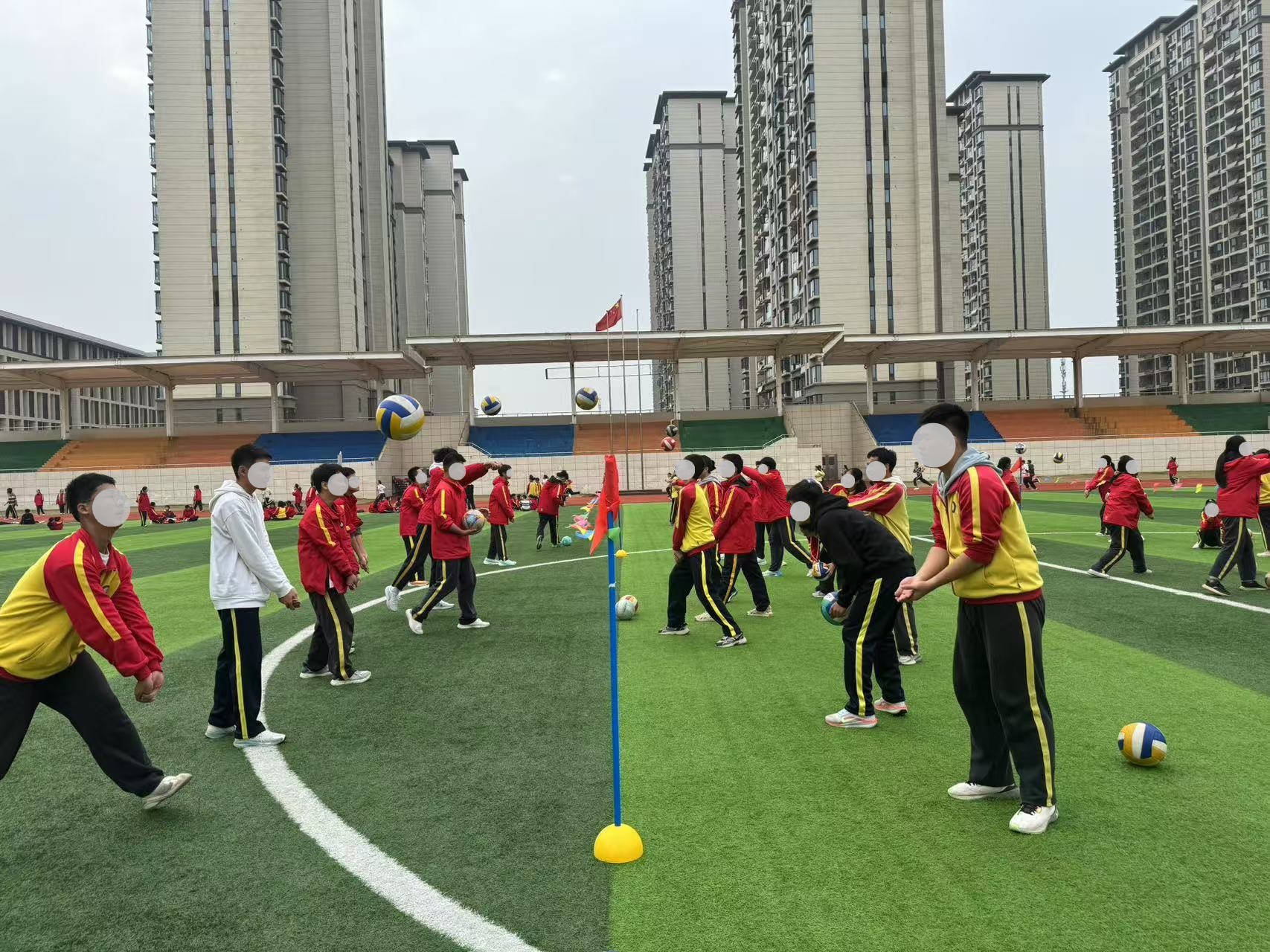

Supplement: S3 Fig — Motor skill exercises conducted in the physical education classroom. (JPG) [file pone.0336894.s007.jpg]

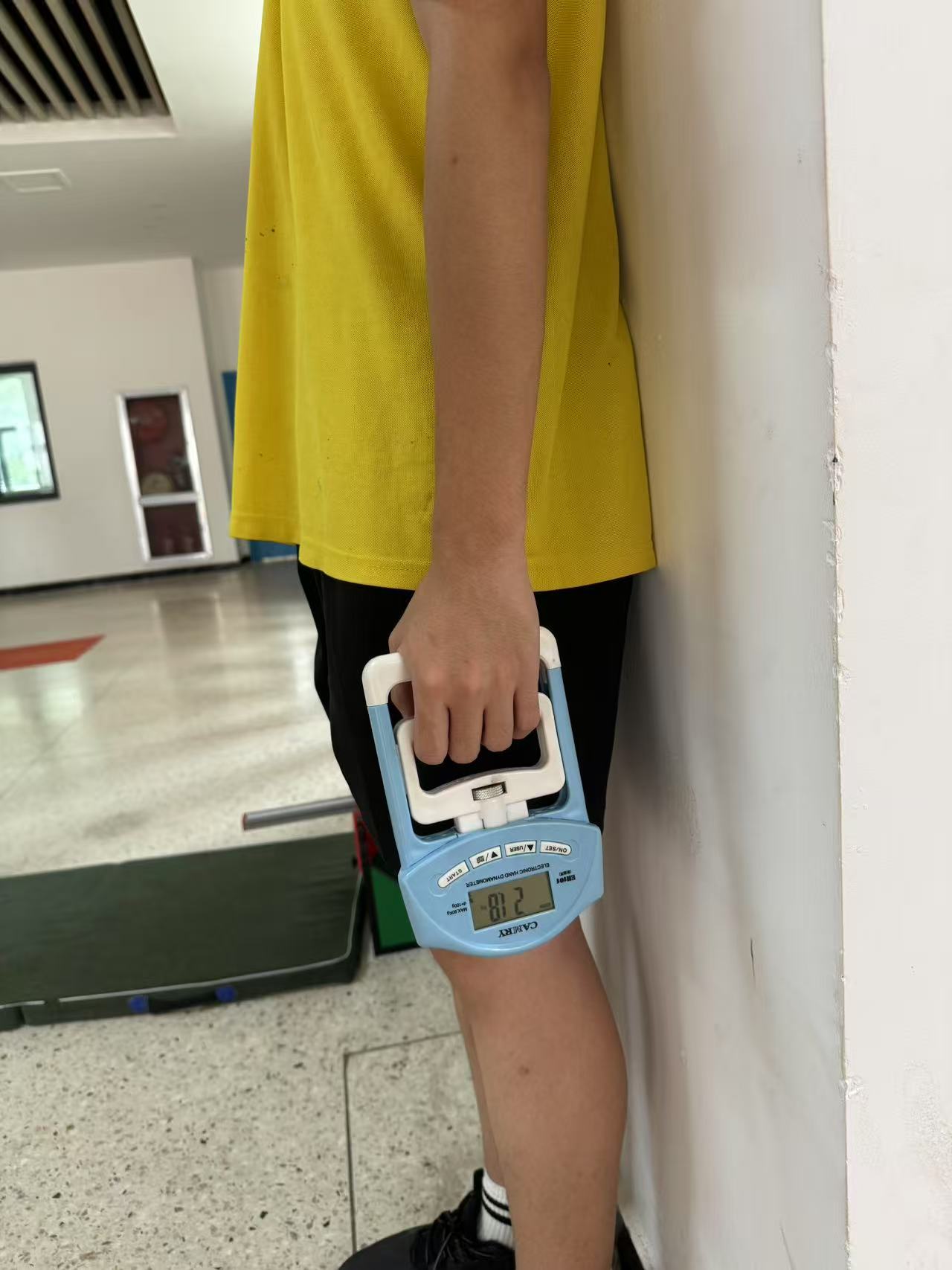

Supplement: S4 Fig — Measurement of handgrip strength. (JPG) [file pone.0336894.s008.jpg]

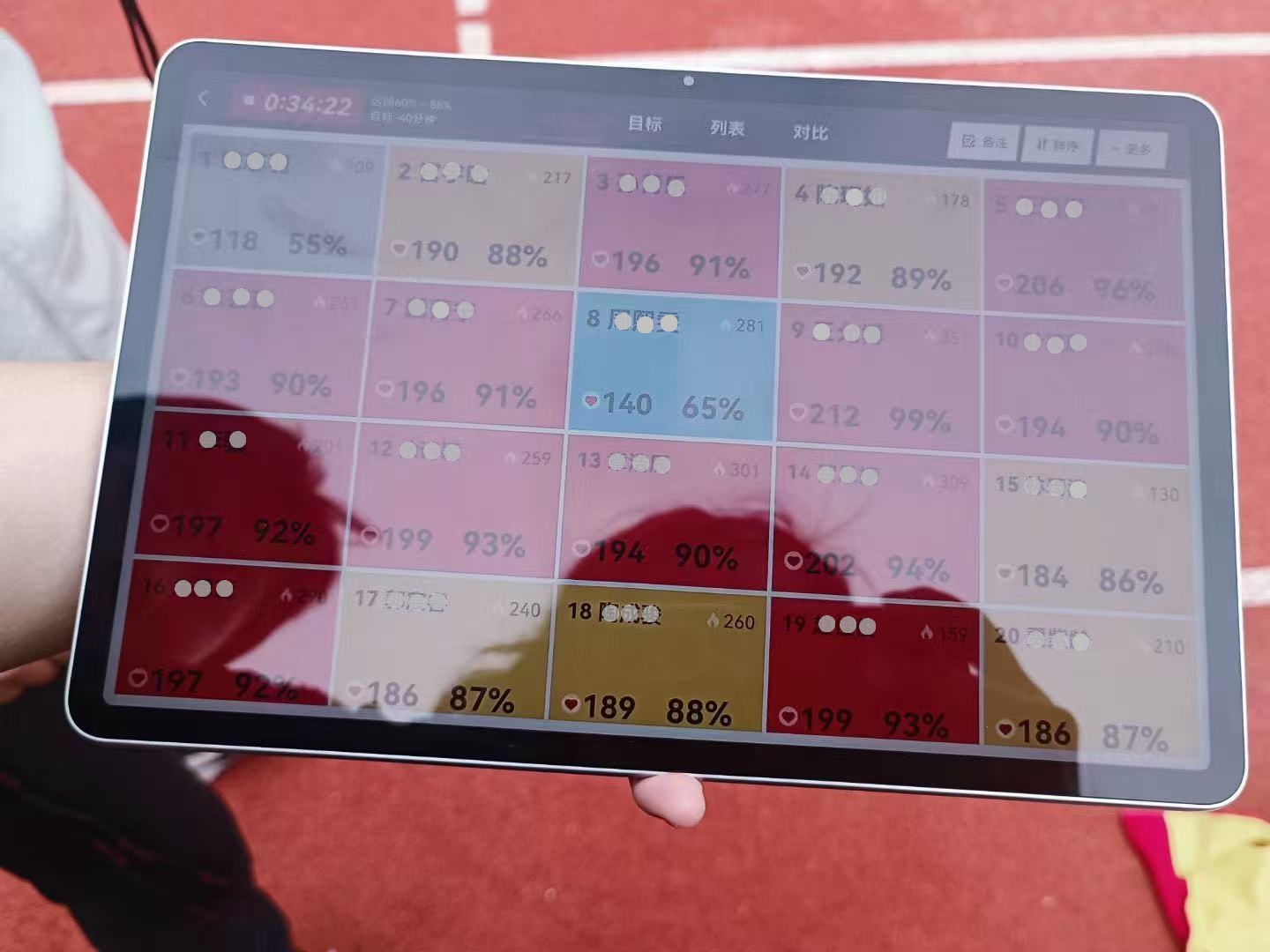

Supplement: S5 Fig — Heart rate monitoring during the physical education class. (JPG) [file pone.0336894.s009.jpg]

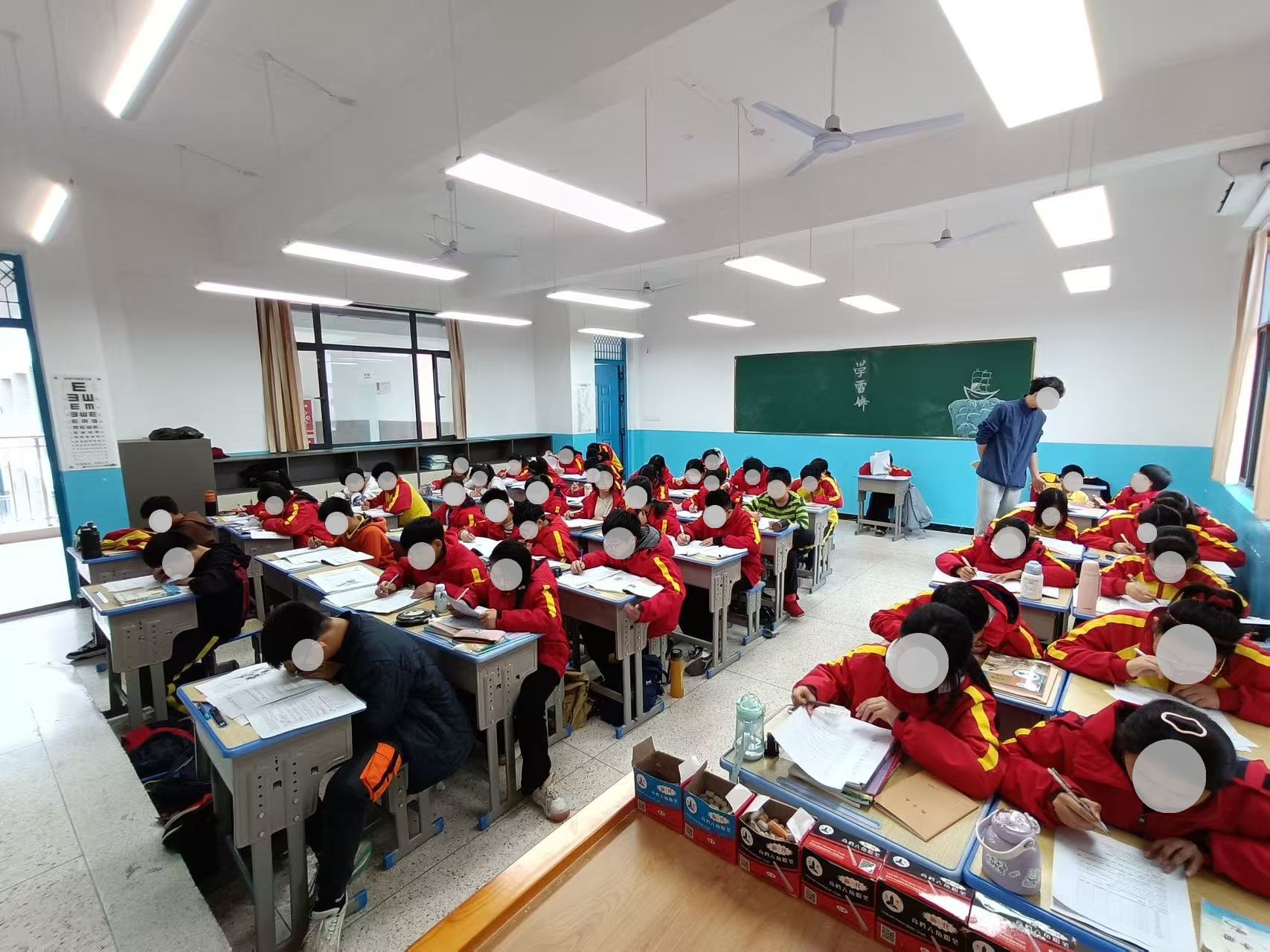

Supplement: S6 Fig — Information collected through a questionnaire. (JPG) [file pone.0336894.s010.jpg]

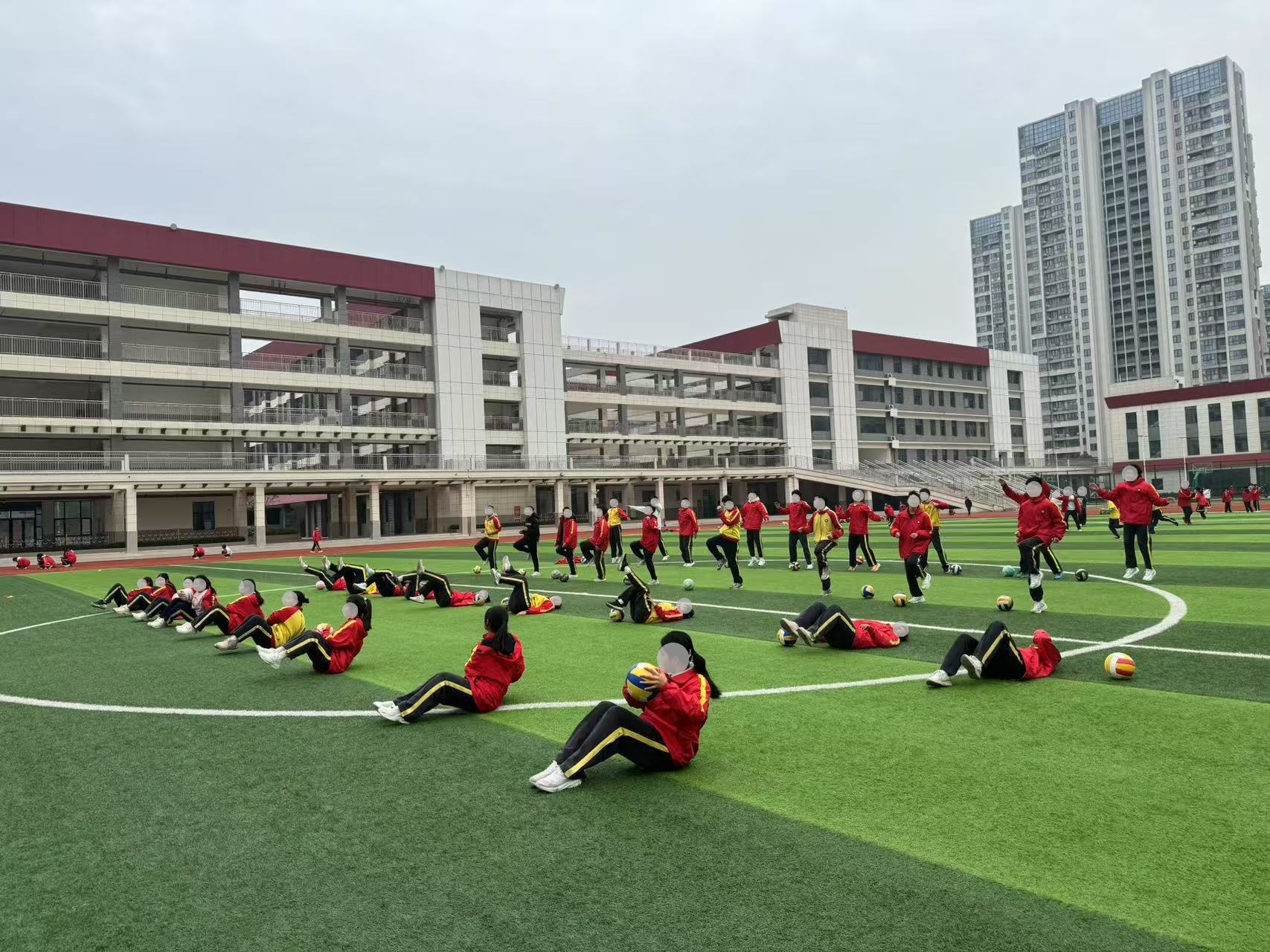

Supplement: S7 Fig — Physical fitness training in the physical education class. (JPG) [file pone.0336894.s011.jpg]

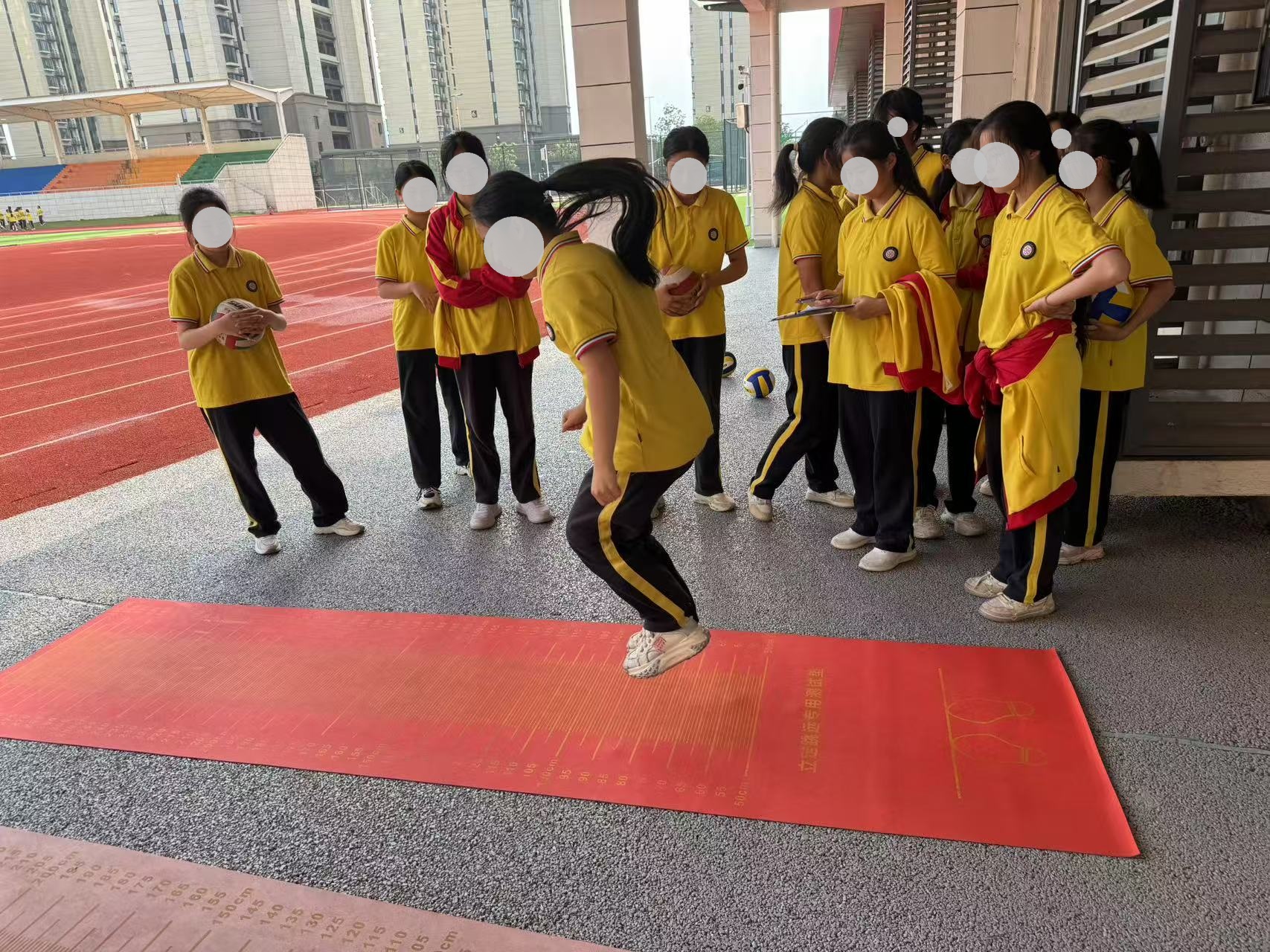

Supplement: S8 Fig — Measurement of standing long jump. (JPG) [file pone.0336894.s012.jpg]
